# Monosomy 21 Seen in Live Born Is Unlikely to Represent True Monosomy 21: A Case Report and Review of the Literature

**DOI:** 10.1155/2014/965401

**Published:** 2014-02-04

**Authors:** Trent Burgess, Lilian Downie, Mark D. Pertile, David Francis, Melissa Glass, Sara Nouri, Rosalynn Pszczola

**Affiliations:** ^1^Victorian Clinical Genetics Services, Murdoch Childrens Research Institute, Royal Children's Hospital, Parkville 3052, Melbourne, Australia; ^2^Sunshine Hospital, Western Health, Sunshine 3020, Melbourne, Australia

## Abstract

We report a case of a neonate who was shown with routine chromosome analysis on peripheral blood lymphocytes to have full monosomy 21. Further investigation on fibroblast cells using conventional chromosome and FISH analysis revealed two additional mosaic cell lines; one is containing a ring chromosome 21 and the other a double ring chromosome 21. In addition, chromosome microarray analysis (CMA) on fibroblasts showed a mosaic duplication of chromosome region 21q11.2q22.13 with approximately 45% of cells showing three copies of the proximal long arm segment, consistent with the presence of a mosaic ring chromosome 21 with ring instability. The CMA also showed complete monosomy for an 8.8 Mb terminal segment (21q22.13q22.3). Whilst this patient had a provisional clinical diagnosis of trisomy 21, the patient also had phenotypic features consistent with monosomy 21, such as prominent epicanthic folds, broad nasal bridge, anteverted nares, simple ears, and bilateral overlapping fifth fingers, features which can also be present in individuals with Down syndrome. The patient died at 4.5 months of age. This case highlights the need for additional studies using multiple tissue types and molecular testing methodologies in patients provisionally diagnosed with monosomy 21, in particular if detected in the neonatal period.

## 1. Introduction

Apparent full monosomy 21 has been reported in ten cases in the pre- and postnatal settings (excluding early pregnancy loss), with most cases being lethal in utero [1–14]. However, many of these cases were reported when cytogenetic techniques were limited and often only single tissues were investigated. Clinical features of monosomy 21 include severe Intrauterine Growth Retardation (IUGR), ear anomalies, clinodactyly (5th finger), seizures, and anteverted nares. Cases of monosomy 21 reported in live born, as was the provisional diagnosis in this case, are unlikely to represent true full monosomy 21. The presence of a second undetected cell line being the most likely explanation for pregnancies reaching term.

The presence of a ring chromosome 21, due to their mitotic instability and propensity for tissue limited mosaicism, provides a plausible explanation for some of the previously reported cases of full monosomy 21. In particular, when detected in the neonatal setting. The associated phenotype in patients with mosaic ring chromosome 21 with monosomy 21 is varied. Features range from apparently normal individuals [15] through to individuals with dysmorphic features and congenital abnormalities [16]. The severity of the phenotype is likely to depend upon the prevalence and tissue distribution of the monosomy 21 cell line, the level of genomic imbalance associated with the formation of the ring chromosome, and the mitotic dynamism of the ring chromosome, also known as “Ring syndrome” [17].

## 2. Case Presentation

The proband, a female infant was the first of dichorionic-diamniotic (DCDA) twin girls born prematurely at 35 + 3 weeks gestation by elective caesarean section for discordant growth on ultrasound. She had a birth weight of 1650 g; her sister weighed 2510 g. This patient was a result of the sixth pregnancy of a nonconsanguineous couple; all other siblings are alive and well. The 35-year-old mother's past medical history was unremarkable except for latent TB infection; her only pregnancy complication was a finding of vitamin D deficiency. She received standard twin pregnancy antenatal care. Initial fetal morphology scans performed at 17 and 21 weeks gestation showed no abnormalities. Discordant growth was first detected at 26 weeks gestation and monitored with serial ultrasound. At 34+ weeks, ultrasound surveillance showed that the first twin's estimated weight had dropped below the third centile while amniotic fluid index and umbilical artery Doppler flows remained within normal limits; short femur length was also noted for the first time at this scan. Due to increasing growth discordance the decision was made for elective delivery at 35 weeks following betamethasone administration.

**Figure 1:**
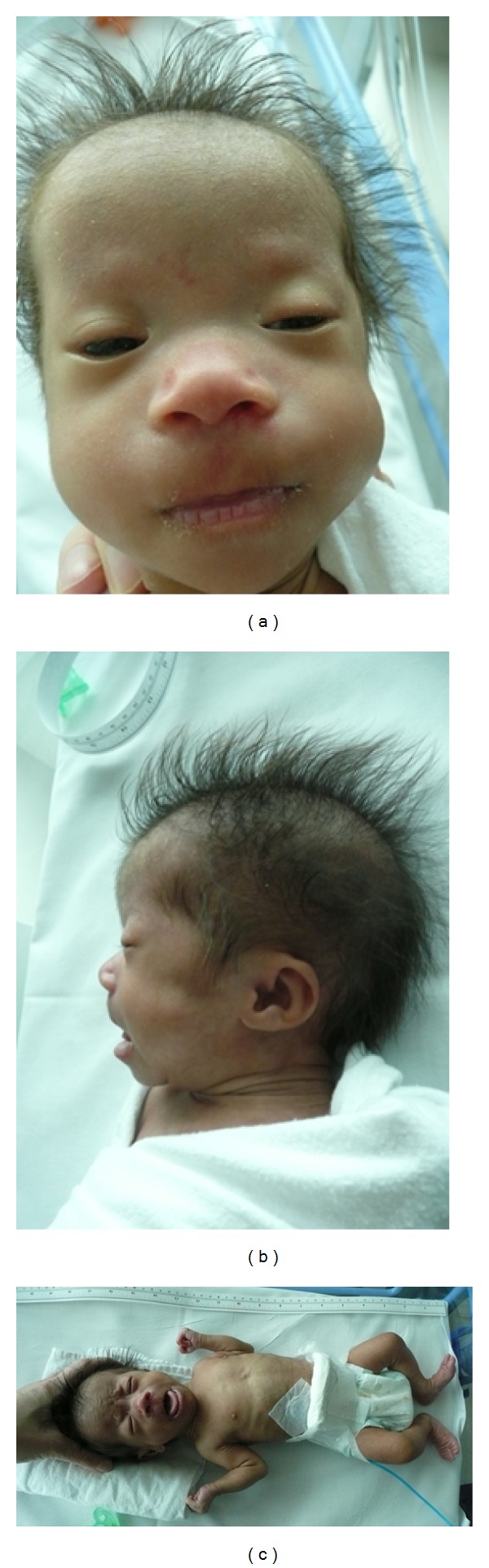
(a) demonstrates the observed broad nasal bridge, anteverted nares, and prominent epicanthis folds, (b) simple ears, and (c) bilateral 5th finger clinodactyly.

The patient required less than one minute of positive pressure ventilation at delivery for poor respiratory effort and was then stabilized on constant positive airway pressure (CPAP). Respiratory support was weaned without complication within the first hour of life. Dysmorphic features were first noted on day 10 of life when she was observed to have prominent epicanthic folds, broad nasal bridge, anteverted nares, simple ears, and bilateral overlapping fifth fingers (Figure 1). Further examinations and imaging were performed to assess for other dysmorphologies and no abnormalities were detected on renal ultrasound, ophthalmology examination, cranial ultrasound, or brain magnetic resolution imaging (MRI). Conventional chromosome analysis on peripheral bloods was requested.

The neonatal course was complicated by seizures occurring on day 22 of life. During these episodes she became pale and inactive and showed jerking movements of all four limbs and had a corresponding decrease in oxygen saturations. Each episode lasted for approximately 20 seconds which self-resolved, but she remained hypotonic for 2-3 minutes following this episode. Treatment was initiated for possible meningitis but colony stimulating factor (CSF) culture and viral studies were negative. No further episodes were noted after this.

A grade 3 systolic cardiac murmur was noted during admission. Subsequent echocardiogram showed a structurally normal heart with a small patent foramen ovale and normal cardiac function.

Persistent thrombocytopenia was documented with platelet nadir of 79 × 10^9^/L on day 25 of life. Blood film analysis was not diagnostic of specific pathology and there was no clinical evidence of bleeding or bruising. All other haematological cell lines were normal.

The patient was discharged on day 63 of life at one month corrected gestational age on full suck feeds with a weight of 2265 grams. On review at 8 weeks corrected age weight gain was very slow with the patient's weight dropping significantly below the 3rd centile in association with poor feeding and decreased caloric. On examination she was markedly hypotonic with significant head lag but demonstrated some antigravity movement in all four limbs. Cardiac murmur was no longer audible.

At 4.5 months the patient died at home after struggling to gain weight. Gavage feeding was discussed but not proceeded with and parents had made a decision not to resuscitate. A postmortem was declined.

## 3. Results and Discussion

A provisional clinical diagnosis of possible trisomy 21 was made on this patient. However, the patient was shown to have full monosomy 21 on a conventional blood karyotype (60 cells analysed) and interphase FISH analysis (100 cells analysed, using the chromosome 21 specific probe provided in the AneuVysion kit (AneuVysion, Abbott Molecular, Illinois, USA)). True full monosomy 21 is rarely observed and is likely to be lethal in utero [18]. Full monosomy 21 has been reported in the miscarriage and prenatal and postnatal settings; however, the rigor of the testing methods in many cases was not adequate to determine conclusively full nonmosaic monosomy 21 [1–14]. This is due to many factors including a lack of molecular cytogenetic protocols available at the time of testing and the unavailability of multiple tissue types for the exclusion of tissue limited mosaicism. Many of the earlier documented cases of full monosomy 21, through retrospective analyses using molecular techniques, have been shown to represent cryptic unbalanced rearrangements and therefore not to represent true monosomy 21. In particular, there have been several reports of unbalanced derivative chromosomes that have resulted from a cryptic translocation between chromosomes 5p and 21q [19–21]. Other cases of full monosomy 21 diagnosed in live born, where unbalanced derivatives chromosomes have been excluded by molecular techniques, often lack an extensive cytogenetic work-up to exclude tissue limited mosaicism [1, 3, 5, 6, 8, 9, 12, 22, 23]. In these cases, only a single or limited number of tissues have been investigated; therefore a cryptic cell line that would act to reduce the level of genomic imbalance has not been excluded. The presence of an undetected cell line would provide a plausible explanation for apparent nonmosaic monosomy 21 conceptus' surviving to term. The patient described by Mori et al. [12] may represent one of the more rigorously investigated cases of full monosomy 21 detected in the new born period. Here, three different tissues were investigated (fetal blood, kidney, and fibroblasts), with all showing full monosomy 21 with cryptic unbalanced rearrangements being excluded. Our patient shared common features with the patient described by Mori et al. [12] such as IUGR in the prenatal period, ear anomalies, and clinodactyly. Our patient also showed features observed in other reports of monosomy 21 such as anteverted nares and seizures in the neonatal period, which may indicate that the observed phenotype in our patient is primarily due to monosomy 21.

**Figure 2:**
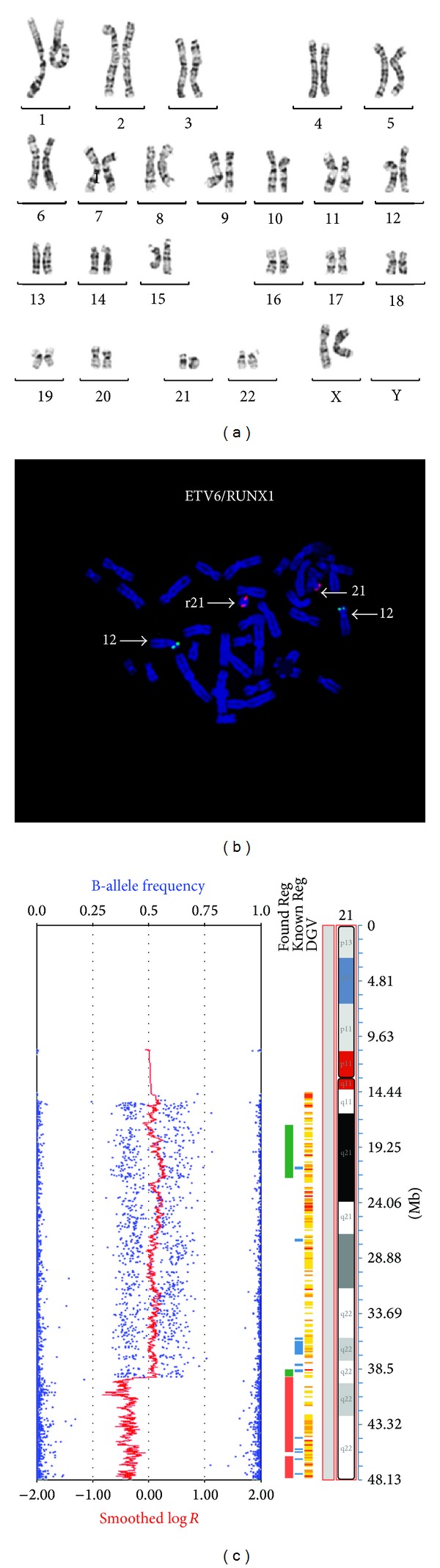
The monosomy 21 cell line is not shown. (a) G-banded fibroblast karyotype showing the ring chromosome 21. (b) Metaphase FISH of cultured fibroblasts using the ETV6/RUNX1 probe set indicating the presence of a double ring, that is, 1 copy of RUNX1 on the normal chromosome 21 and 2 copies on the double ring chromosome 21. (c) Chromosome microarray using the Illumina HumanCoreExome v1 performed on cultured fibroblast showing a copy number (Log⁡ *R*) that is consistent with the presence of a monosomic cell line and cell lines with a ring and double ring chromosome 21. The 8.8 Mb terminal deletion is also indicated. The B-allele frequency also confirms the mosaic nature of this finding.

Interphase FISH analysis on buccal cells using the same chromosome 21 specific AneuVysion FISH probe as used on the blood investigation also showed full monosomy 21. However, conventional chromosome analysis on cultured fibroblasts revealed a mosaic karyotype with two cell lines, one with monosomy 21 (8 cells) and the other with a ring chromosome 21 (42 cells). The karyotype is as follows: 45,XX,-21[8]/46,XX,r(21)(p11q?22)[42] (Figure 2(a), only the ring 21 cell line is shown). The detection of this cryptic mosaic ring chromosome 21 cell line is likely to be the reason that our patient survived until 4.5 months of age. The presence of cryptic cell lines that act to reduce the level of genomic imbalance may also provide a plausible explanation for previously reported cases of full monosomy 21 surviving into the neonatal period. Ring chromosomes are known for their mitotic instability and tissue limited mosaicism, so they may not always be detected by investigating a single or small number of tissues. Ring chromosome 21 is a relatively uncommon anomaly and has been described in both the familial and de novo settings [24]. It is associated with a variable phenotype ranging from marked dysmorphism, developmental delay, and death in early infancy to entirely normal growth, development, and appearance [15, 16, 25, 26]. This variability in phenotype is likely to be due to the prevalence and tissue distribution of the monosomy 21 and ring chromosome 21 cell lines, the genomic imbalance (deletions and duplications) associated with ring chromosome formation, and the level of mitotic dynamism “Ring syndrome” observed within the carrier. Investigation of additional tissues (skin fibroblasts and buccal cells) in our patient revealed the presence of a mosaic ring chromosome 21 in the fibroblast cells, highlighting the importance of further investigation in patients who have been found to have full monosomy 21 in a primary tissue sampling.

Further complicating the phenotype of patients with mosaic or nonmosaic ring chromosome 21 is the level of genomic imbalance associated with the ring chromosome formation. Microarray analysis using the Illumina HumanCoreExome v1 (Illumina, San Diego, CA, USA) on cultured skin fibroblasts in our patient confirmed the presence of the mosaic ring chromosome 21 and also showed a terminal long arm deletion of 8.8 Mb (Figure 2(c)). The combined conventional and molecular karyotypes were determined as 45,XX,-21[8]/46,XX,r(21)(p11q?22)[42].arr 21q11.2q22.13 (15,396,340-39,267,060) x2~3, 21q22.13q22.3 (39,270,074-48,084,247) x1. The deletion, containing approximately 96 genes, resulted in complete monosomy for this region (chr21:39,270,074-48,084,247, UCSC Genome build February 2009 GRCh37/hg19). In a review of terminal chromosome 21 deletions, Lyle et al. [27] suggested that terminal deletions in the range of 5.6 Mb to 11 Mb (comparable to our patient) show a relatively mild phenotype, which includes moderate mental retardation and may or may not include subtle dysmorphisms. It is highly likely that the loss of the 8.8 Mb segment has contributed to our patients phenotype; however, it may be that it has had a milder impact on the phenotype than the loss of the ring chromosome in the monosomy 21 cell line.

In addition, the presence of the relatively large terminal deletion in our case had the potential to cause confusion when investigating the various tissues for monosomy 21. The commercially available and widely used AneuVysion chromosome 21 specific probe, which maps to the proposed Down Syndrome Critical Region (DSCR) at 21q22.13, was contained within the deleted region (by 100 Kb) and would always give the appearance of full monosomy 21 in tissues where the ring chromosome 21 was present. We are confident that the blood sample only contained full monosomy 21 as the microarray performed on DNA extracted from whole blood did not show any evidence of mosaicism for ring chromosome 21 (sensitivity to 5% mosaicism and above as determined by an internal laboratory spiking experiment). However, the presence of the ring chromosome 21 could not be excluded in the buccal sample, which showed full monosomy 21 using FISH (AneuVysion), as no confirmatory testing could be performed. Metaphase FISH using the ETV6/RUNX1 probe (Abbott Molecular, Illinois, USA), which is located outside the deleted region, was performed on cultured fibroblast. This investigation showed 1 copy of RUNX1 (chromosome 21 derived) in thirty cells (31%), 2 copies in twenty-nine cells (30%), and 3 copies in thirty-eight cells (39%), indicating the presence of the monosomy cell line, the cell line with the ring chromosome, and an additional cell line with a double sized ring (Figure 2(b), only double ring shown). This highlights one of the many benefits CMA has provided in the investigation of patients suspected of having a chromosome abnormality, allowing for a more accurate evaluation of the genomic imbalance and directing more appropriate follow-up testing protocols.

The mitotic instability observed in our patient resulted in a cell line that contained double rings, meaning there was mosaicism for partial trisomy. In the fibroblast sample the double ring was present in approximately 39% of cells. The high level of double rings in some tissues in our patient may have contributed to the patient's provisional clinical diagnosis of Down syndrome when the ring chromosome 21 is larger in size (i.e., less genomic imbalance due to monosomy) and demonstrates duplication (such as double rings) that includes the DSCR; the patient is more likely to have characteristics consistent with Down syndrome [28]. However, the phenotype of patients previously reported as full monosomy 21 appears to share features common to Down syndrome, such as heart defects, clinodactyly, simian crease, and upslanting palpebral fissures [1–3, 5, 8–10, 12, 13, 23]. As discussed, however, the presence of an additional cell line has not been excluded in many of these previously reported cases.

Ring chromosomes have been observed for all human chromosomes and are well known to be associated with abnormal phenotypes. The impact on phenotype is proposed to be the result of either loss/gain of genetic material during the ring formation (as discussed above) and/or mitotic dynamism of the ring chromosome [17, 29]. The latter can result in cell lines that do not contain the ring (monosomy), cells that contain the ring, and cells with double sized rings (trisomy). Double ring chromosomes have been shown to result from sister chromatid exchange events. This mitotic instability is proposed to lead to increased cell mortality and result in severe growth deficiencies, this being the key feature of “Ring syndrome” [17]. It is difficult to ascertain the significance that “Ring syndrome” may have played in the patient in this case report, especially given the high frequency of the monosomy cell line and the size of the terminal deletion; however it is likely that this has contributed to the evolution of the three cell lines observed.

In conclusion, full monosomy 21 is likely to be lethal in utero. Patients who have been found to have full monosomy 21 in the neonatal period require further investigations using a combination of conventional and molecular cytogenetics techniques to exclude cryptic unbalanced rearrangements. In addition, where available, multiple tissue lineages need to be tested to determine the presence of a cryptic cell line that would act to reduce the level of genomic imbalance. An additional cell line was confirmed in the patient in this case report which constituted a ring chromosome 21. The ring chromosome was shown to have an 8.8 Mb terminal deletion and was shown to be mitotically unstable. CMA provided an important new cytogenetic tool, allowing for the accurate determination of genomic imbalance, the level of mosaicism, and the appropriate selection of FISH probes for interphase FISH examinations, and aided in providing more accurate prognostic information.
